# Molecular allergen sensitization of *Aspergillus fumigatus* between allergic bronchopulmonary aspergillosis and *A fumigatus*‐sensitized asthma in Guangzhou, Southern China

**DOI:** 10.1002/jcla.23448

**Published:** 2020-07-02

**Authors:** Wenting Luo, Haisheng Hu, Zehong Wu, Nili Wei, Huimin Huang, Peiyan Zheng, Yong Liu, Baoqing Sun

**Affiliations:** ^1^ Department of Allergy and Clinical Immunology State Key Laboratory of Respiratory Disease National Clinical Research Center for Respiratory Disease Guangzhou Institute of Respiratory Health The First Affiliated Hospital of Guangzhou Medical University Guangzhou China; ^2^ Department of Immunology Guangzhou Kingmed Diagnostics Group Co., Ltd. Guangzhou China

**Keywords:** allergic bronchopulmonary aspergillosis, *Aspergillus fumigatus*, *Aspergillus fumigatus*‐sensitized asthma, molecular allergen, mycotic allergens

## Abstract

**Background:**

Few studies have assessed the sensitization of mycotic allergens and *Aspergillus fumigatus* molecular allergens. This study aimed to investigate the relationships of *A fumigatus* components and mycotic allergens in allergic bronchopulmonary aspergillosis (ABPA) patients and *A fumigatus* (Af)‐sensitized asthma patients.

**Methods:**

Serum sIgE levels of *Penicillium chrysogenum*, *Cladosporium herbarum*, *Mucor racemosus*, *Candida albicans*, *Alternaria alternata*, *Helminthosporium halodes,* and *A fumigatus* allergen components (Asp f 1, Asp f 2, Asp f 3, Asp f 4, and Asp f 6) were measured via the ImmunoCAP assay in 18 ABPA and 54 Af‐sensitized asthma patients in Guangzhou city, China.

**Results:**

94.44% of ABPA patients and 87.04% of Af‐sensitized asthma patients were co‐sensitized to at least one other fungal allergen. The positive rates of Asp f 1 (88.89% vs 59.26%, *P* < .05), Asp f 2 (66.67% vs 33.33%, *P* < .05), Asp f 4 (61.11% vs 33.33%, *P* < .05), and Asp f 6 (66.67% vs 14.81%, *P* < .001) in ABPA patients were higher than those in Af‐sensitized asthma patients. IgE levels of Asp f 1 (*P < *.05), Asp f 4 (*P < *.05), and Asp f 6 (*P < *.001) were higher in ABPA patients than in Af‐sensitized asthma patients. Optimal scale analysis showed that ABPA was more relevant to Af components (Cronbach's alpha = 90.7%).

**Conclusion:**

The *A fumigatus* components and their relationships with various mycotic allergens were different in ABPA and Af‐sensitized asthma patients. This finding may help local doctors in the diagnosis and immunotherapy of fungal allergies.

## Graphical abstract

Patients with ABPA were characterized by higher levels of IgE antibodies to Asp f 1, Asp f 4, and Asp f 6 than those of Af‐sensitized asthma patients (all*P* < .05). The combination of sensitized to all five component allergens of *Aspergillus fumigatus* were higher in patients with ABPA (36.84%) than that in patients with asthma (18.52%).

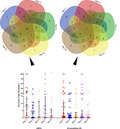

## 1 INTRODUCTION

Fungi are the most common microorganisms in the human living environment, and they not only readily cause respiratory tract infections and induced inflammatory responses but also cause severe allergic reactions. Studies have shown that *Aspergillus*, *Alternaria*, *Candida*, *Cladosporium*, and *Epicoccum* are considered major sources of allergens worldwide.^1^, ^2^ During reproduction, fungi release large amounts of spores and hyphal fragments into the air, which may cause immunoglobulin E (IgE)‐mediated respiratory allergic diseases,^3^ especially *Aspergillus fumigatus* (Af)‐sensitized asthma and allergic bronchopulmonary aspergillosis (ABPA).^4^ Investigations in Europe have shown that the incidence of fungus‐induced respiratory tract allergies is as high as 20%‐30% in atopic populations, reaching 6% in the general population.^5^, ^6^

Allergic bronchopulmonary aspergillosis is a pulmonary disease caused by *A fumigatus*, and pathogenesis is caused by the allergic response against *A fumigatus* colonizing the airways rather than saprophytic or invasive of the fungi.^7^, ^8^ When patients with ABPA are exposed to fungi in the environment, they display repeated wheezing and dyspnea; this condition can even be life‐threatening in severe cases.^9^ Due to the lack of effective clinical treatment, patients have heavy economic burden and poor quality of life. A European study showed that Af‐sensitized asthma can easily develop into ABPA,^10^, ^11^ which may be induced by molecular allergens of *A fumigatus.* There are five major molecular allergens of *A fumigatus* (Asp f 1, Asp f 2, Asp f 3, Asp f 4, and Asp f 6). Among them, Asp f 1 is the most important protein of *A fumigatus*. It secretes a lot after spore germination and early fungal invasion, which is related to fungal colonization and the saprophytic nature of the fungi. Asp f 2 is a fibronectin, Asp f 3 is an epitope of the peroxidase membrane protein, Asp f 4 is a glycosylated hydrolase, and Asp f 6 is a manganese superoxide dismutase.^12^, ^13^ Our previous research showed that more than 18% of asthmatic patients are sensitized to Asp f 3.^14^

Although several studies have focused on *A fumigatus* sensitization in Southern China,^15^, ^16^ the studies investigating the connection between various fungal allergens and *A fumigatus* components are still limited, especially in Guangzhou, a beautiful and unique cultural city in China. In addition, various fungal allergens and *A fumigatus* components may be co‐pathogenic and play an important role in ABPA or Af‐sensitized asthma. Accordingly, in this study, we compared various fungal allergens and *A fumigatus* major components between ABPA and Af‐sensitized asthma patients, and our findings are expected to provide meaningful evidence for more accurate diagnosis and guiding disease treatment.

## 2 MATERIALS AND METHODS

### 2.1 Patients

This study included 18 ABPA patients and 54 Af‐sensitized asthma patients, all of whom had undergone *A fumigatus* allergen sIgE tests between January 2016 and December 2017 in the Allergy Information Repository of the National Clinical Research Center for Respiratory Disease (AIR‐NCRCRD, Guangzhou, Southern China). The inclusion criteria for Af‐sensitized asthma patients were as follows: (a) clinical diagnosis of asthma; (b) allergic clinical symptoms following contact with fungal allergen, such as wheezing, dyspnea, and/or chronic cough not caused by a cold; (c) *A fumigatus* sIgE ≥ 0.35 kUA/L; and (d) tIgE < 1000.00 kUA/L. The diagnosis of asthma was based on the Global Initiative for Asthma guidelines. The diagnosis of asthma was based on the Global Initiative for Asthma guidelines,^17^ and the diagnostic criteria of ABPA were based on Agarwal et al^18^ by a respiratory specialist. Patients with a history of allergen‐specific immunotherapy, parasitic infections, cancer, and immunodeficiency were excluded. All patients provided written informed consent. There were no significant differences in age and sex of patients between the groups. Approval was obtained from the ethics committee of The First Affiliated Hospital of Guangzhou Medical University (Reference number: GYFYY‐2016‐73).

### 2.2 Serum allergen‐specific IgE detection

In 18 patients with ABPA and 54 patients with Af‐sensitized asthma, serum sIgE levels of *Penicillium chrysogenum*, *Cladosporium herbarum*, *Aspergillus fumigatus*, *Mucor racemosus*, *Candida albicans*, *Alternaria alternata*, *Helminthosporium halodes*, total IgE, and *Aspergillus fumigatus*components (Asp f 1, Asp f 2, Asp f 3, Asp f 4, and Asp f 6) were tested by PhadiaCAP 1000 (Thermo Fisher Scientific, Göteborg, Sweden). SIgE concentrations of 0.35 kUA/L or more were defined as positive or sensitized to the allergen. According to the sIgE levels, the reactivity was categorized quantitatively into six classes: Class 1 (≥0.35 kUA/L to <0.70 kUA/L), Class 2 (≥0.70 kUA/L to <3.50 kUA/L), Class 3 (≥3.50 kUA/L to <17.50 kUA/L), Class 4 (≥17.50 kUA/L to <50.00 kUA/L), Class 5 (≥50.00 kUA/L to <100.00 kUA/L), and Class 6 (≥100.00 kUA/L).^19^

### 2.3 Statistical analyses

Data analyses were performed using the statistical software package SPSS 22.0 (Chicago, IL, USA). Nonparametric quantitative data were described as medians (interquartile ranges) and between‐group comparisons of numerical data were performed using Mann‐Whitney *U* tests or Kruskal‐Wallis tests. Parametric quantitative data were depicted as means ± standard deviations. To show the proportion of positive results, categorical data were reported as percentages. Chi‐square (*χ* ^2^) tests or *F* tests were used to demonstrate differences in proportions between groups. Correlation analyses among the groups were performed by calculating the Spearman correlation coefficient (*r_s_*). The correlation between components was calculated with optimal scale analysis. Differences were regarded as statistically significant if the *P* value was lower than .05.

## 3 RESULTS

### 3.1 Fungal sensitization between ABPA patients and Af‐sensitized asthma patients

**Figure 1:**
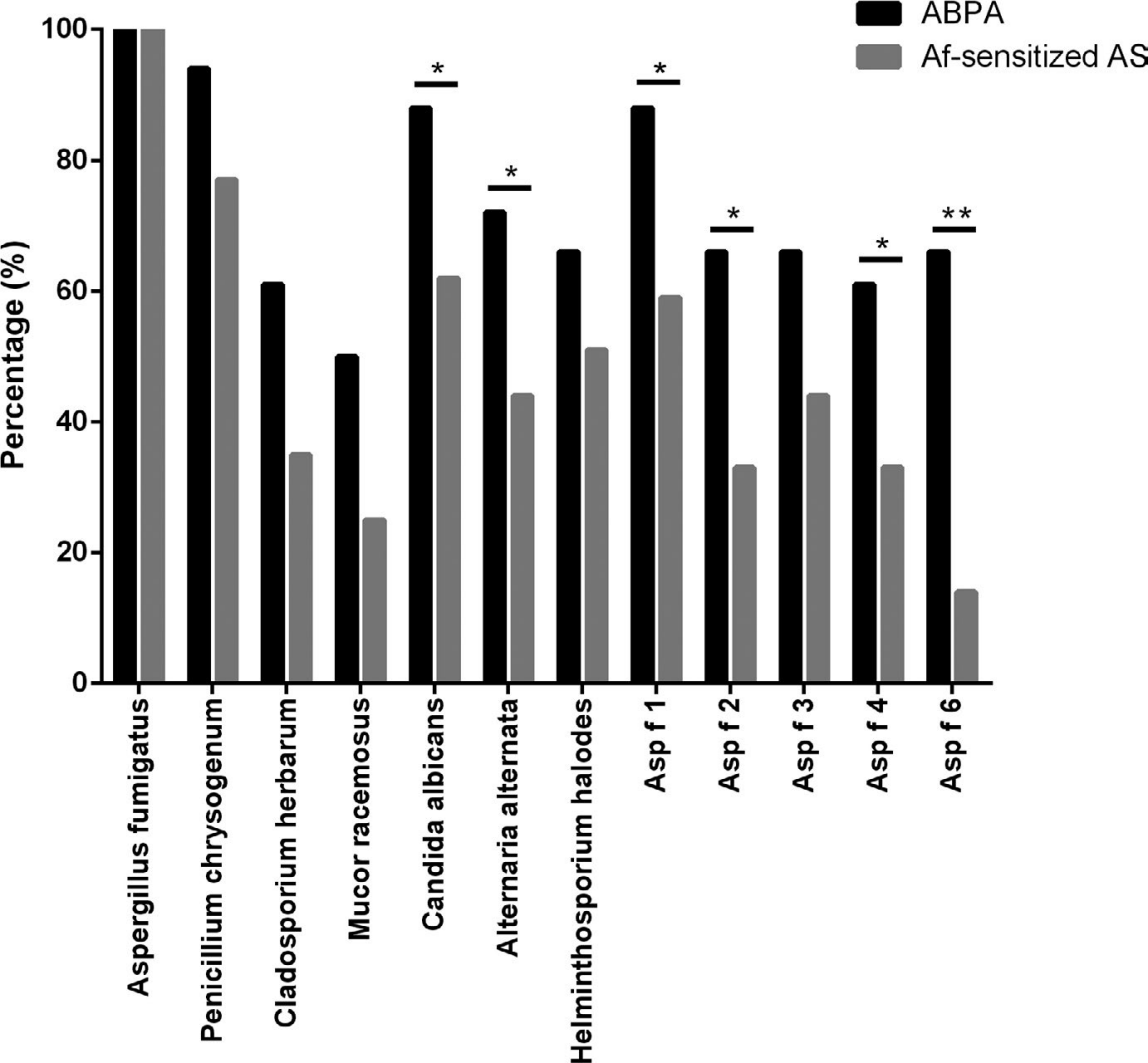
The positive rate of fungal allergens and *Aspergillus fumigatus* components between ABPA and *A fumigatus‐*sensitized asthma patients. ABPA, allergic bronchopulmonary aspergillosis; Af‐sensitized, AS, *A fumigatus‐*sensitized asthma patients

**Figure 2:**
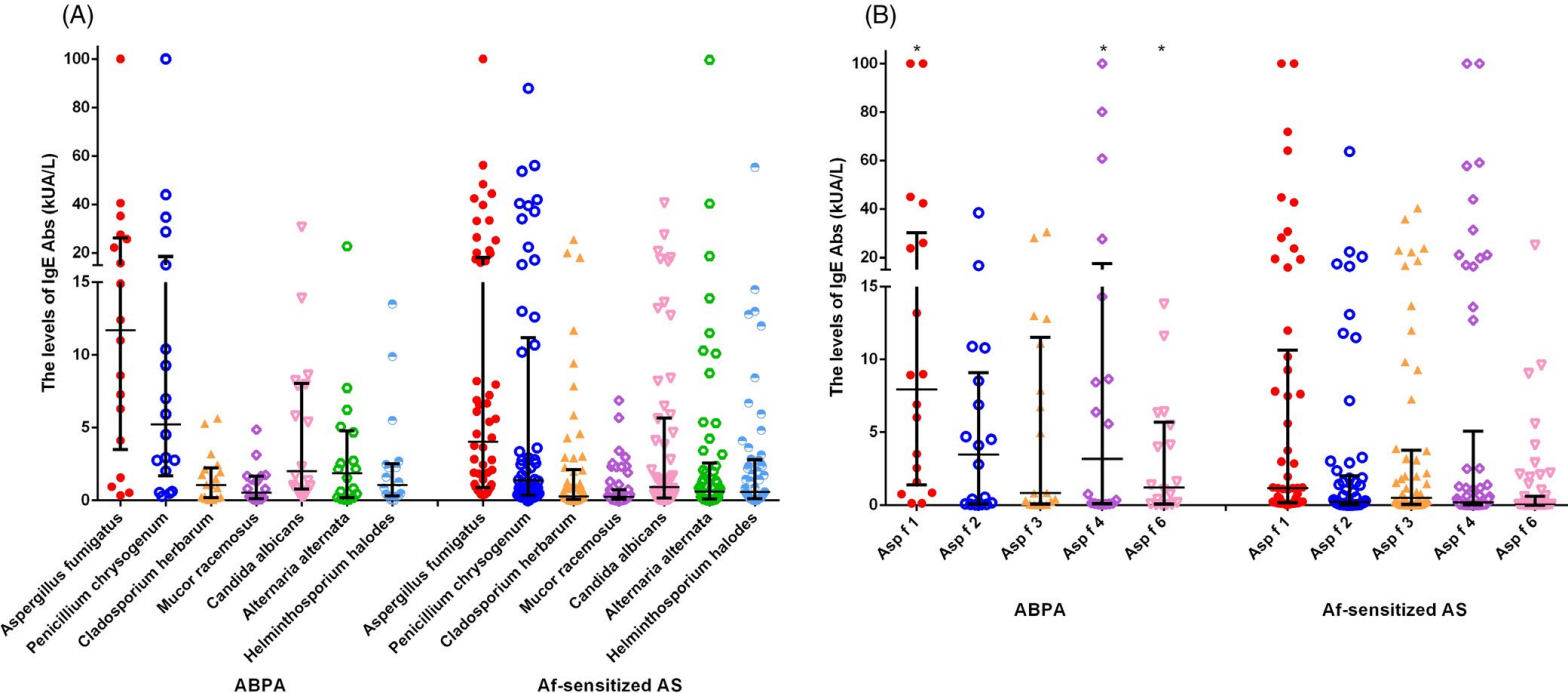
The sIgE levels of fungal allergens and *Aspergillus fumigatus* components between ABPA and *A fumigatus‐*sensitized asthma patients. (a) Fungal allergens; (b) *A fumigatus* components. ABPA, allergic bronchopulmonary aspergillosis; Af‐sensitized, AS, *A fumigatus‐*sensitized asthma patients

Overall, 31.9% of patients were sensitive to *A fumigatus* in Class 3. (Table 1). There was no significant difference in *A fumigatus* sIgE levels between ABPA and Af‐sensitized asthma patients (*P > *.05). Moreover, 94.44% of ABPA patients and 87.04% of Af‐sensitized asthma patients were sIgE positive to at least one fungal allergen among *P chrysogenum*, *C herbarum*, *M racemosus*, *C albicans*, *A alternata*, and *H halodes*. High positive rates to *P chrysogenum* were found in 94.44% of ABPA patients and 77.78% of Af‐sensitized asthma patients. The positivity rates of *C herbarum* (88.89% vs 62.96%, *P < *.05) and *A alternata* (72.22% vs 44.44%, *P < *.05) were higher in ABPA patients than in Af‐sensitized asthma patients (Figure 1). Although sIgE levels of *P chrysogenum*, *C herbarum*, *M racemosus*, *C albicans*, *A alternata*, and *H halodes* were higher in ABPA patients than in Af‐sensitized asthma patients, there were no significant differences between the two groups (Figure 2A).

**Table 1:** *Aspergillus fumigatus* sensitization classes in the two groups

Characteristic (n, %)	Af‐sensitized asthma	ABPA
Total	54	18
Sex		
Female	20, 37.0%	11, 61.1%
Male	34, 63.0%	7, 38.9%
Age		
≤18 years	25, 46.3%	8, 44.4%
>18 years	29, 53.7%	10, 55.6%
sIgE class		
Class 1	9, 16.7%	2, 11.1%
Class 2	16, 29.6%	2, 11.1%
Class 3	15, 27.7%	8, 44.4%
Class 4	12, 22.2%	5, 27.8%
Class 5	1, 1.9%	0, 0.0%
Class 6	1, 1.9%	1, 5.6%

Note

Class 1 (≥ 0.35 kUA/L to < 0.70 kUA/L), Class 2 (≥ 0.70 kUA/L to < 3.50 kUA/L), Class 3 (≥ 3.50 kUA/L to < 17.50 kUA/L), Class 4 (≥ 17.50 kUA/L to < 50.00 kUA/L), Class 5 (≥ 50.00 kUA/L to < 100.00 kUA/L), and Class 6 (≥ 100.00 kUA/L).

### 3.2 A fumigatus component sensitization between ABPA patients and Af‐sensitized asthma patients

The positive rates of Asp f 1 (88.89% vs 59.26%, *P < *.05), Asp f 2 (66.67% vs 33.33%, *P < *.05), Asp f 4 (61.11% vs 33.33%, *P < *.05), and Asp f 6 (66.67% vs 14.81%, *P < *.001) in ABPA patients were significantly higher than those in Af‐sensitized asthma patients.

**Figure 3:**
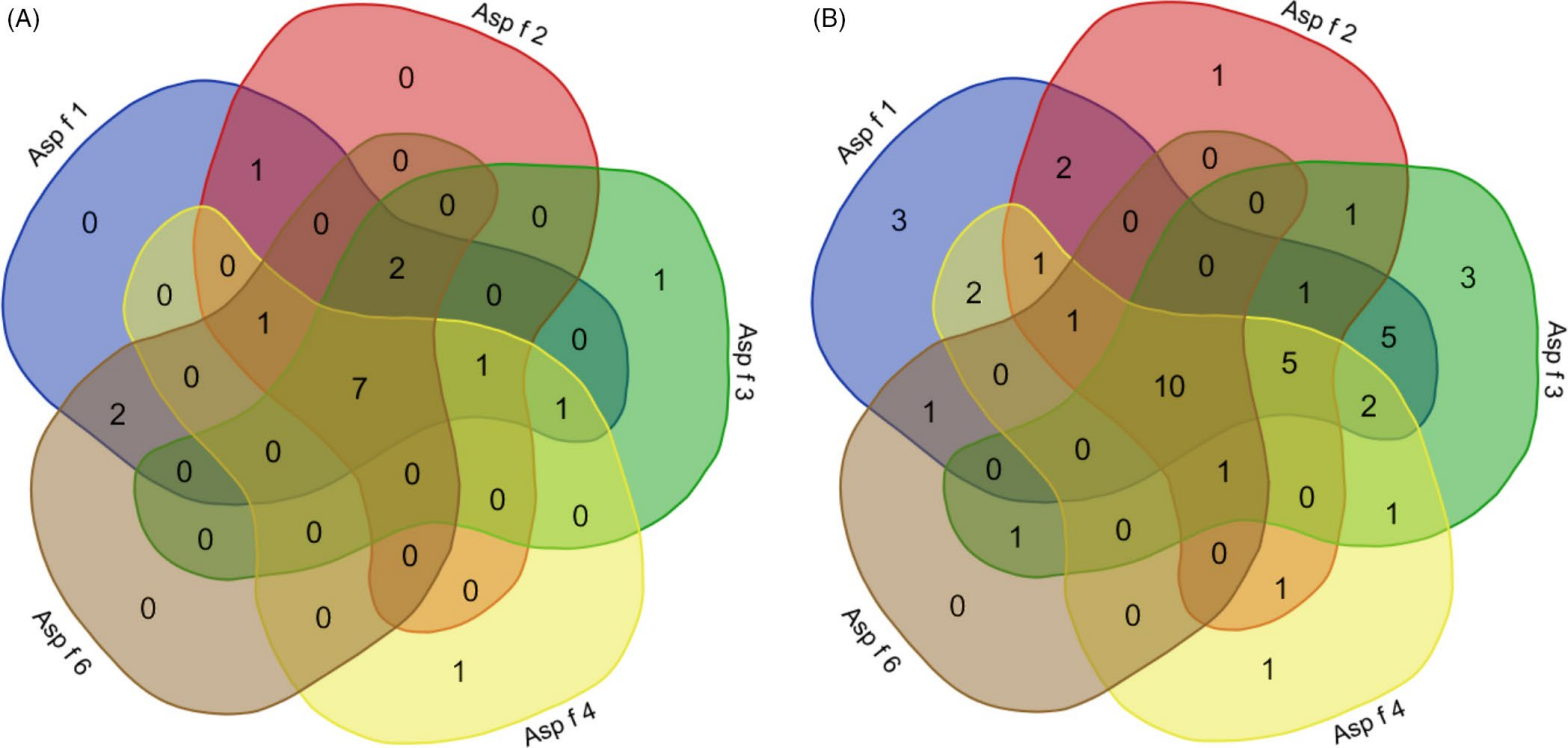
Co‐sensitization of *Aspergillus fumigatus* components between ABPA and *A fumigatus‐*sensitized asthma patients. (a) Allergic bronchopulmonary aspergillosis; (b) *A fumigatus‐*sensitized asthma patients

As shown in Figure 2B, sIgE levels of Asp f 1 [7.93 (1.40, 30.18) kUA/L vs. 0.18 (1.18, 10.65) kUA/L, *P < *.05], Asp f 4 [3.17 (0.10, 17.65) kUA/L vs. 0.03 (0.18, 5.07) kUA/L, *P < *.05], and Asp f 6 [1.22 (0.07, 5.70) kUA/L vs. 0.01 (0.05, 0.61) kUA/L, *P < *.001] in ABPA patients were higher than those in Af‐sensitized asthma patients. The co‐sensitization of five allergen components is shown in Figure 3; there were 7 (36.84%) ABPA patients (the sIgE level of 7 fungal allergens were showed in Table 2) and 10 (18.52%) Af‐sensitized asthma patients co‐sensitized to Asp f 1, Asp f 2, Asp f 3, Asp f 4, and Asp f 6 at the same time. Interestingly, all of the 7 ABPA patients were co‐sensitized to *P chrysogenum* and *C albicans*, but Af‐sensitized asthma patients were not.

**Table 2:** The sIgE levels of various mycotic allergens in ABPA patients which were co‐sensitized to Asp f 1, Asp f 2, Asp f 3, Asp f 4, and Asp f 6

No. (kU/L)	*Penicillium chrysogenum*	*Cladosporium herbarum*	*Aspergillus fumigatus*	*Mucor racemosus*	*Candida albicans*	*Alternaria alternata*	*Helminthosporium halodes*
1	28.80	5.28	35.30	1.74	7.86	7.72	13.5
2	9.28	2.46	15.90	0.80	8.23	5.07	2.28
3	10.40	1.08	6.30	1.46	1.72	2.72	1.27
4	100.00	1.49	12.40	4.86	5.38	2.56	1.61
5	44.10	1.09	27.60	3.13	8.62	2.18	2.49
6	5.93	1.95	40.60	0.21	1.00	2.15	0.50
7	2.76	0.16	8.61	0.09	0.46	0.11	0.31

### 3.3 Correlation analysis between A fumigatus components and various mycotic allergens in ABPA patients and Af‐sensitized asthma patients

**Figure 4:**
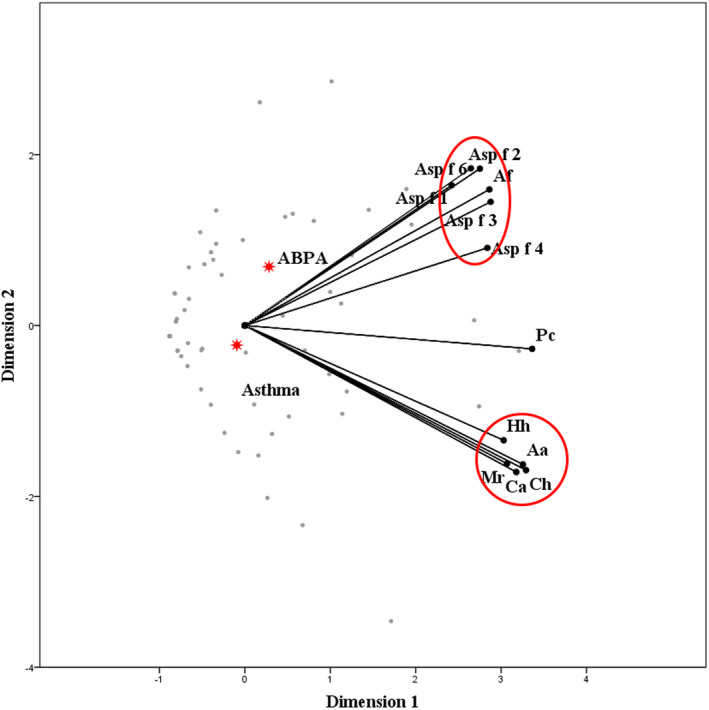
The optimal scale analysis of fungal allergens and *Aspergillus fumigatus* components. The closer the distance between points, the closer the relationship is. Compared to Af‐sensitized asthma patients, ABPA was more relevant to Af components. (Cronbach's alpha = 90.7%). Pc, *Penicillium chrysogenum*; Ch, *Cladosporium herbarum*; Af, *Aspergillus fumigatus*; Mr, *Mucor racemosus*; Ca, *Candida albicans*; Aa, *Alternaria alternata*; Hh, *Helminthosporium halodes*

Spearman correlation analysis showed that tIgE (*r_s_* = 0.586, *P* < .05), *P chrysogenum* (*r_s_* = 0.686, *P* < .05), *C herbarum* (*r_s_* = 0.688, *P* < .05), *M racemosus* (*r_s_* = 0.358, *P* < .05), *C albicans* (*r_s_* = 0.492, *P* < .05), *A alternata* (*r_s_* = 0.692, *P* < .05), and *H halodes* (*r_s_* = 0.585, *P* < .05) sIgE levels were correlated with that of *A fumigatus*. SIgE levels of *A fumigatus* components Asp f 1 (*r_s_* = 0.473, *P* < .05), Asp f 2 (*r_s_* = 0.553, *P* < .05), Asp f 3 (*r_s_* = 0.558, *P* < .05), Asp f 4 (*r_s_* = 0.646, *P* < .05), and Asp f 6 (*r_s_* = 0.730, *P* < .05) were correlated with tIgE. In addition, sIgE levels of Asp f 2 (*r_s_* = 0.653, *P* < .05), Asp f 3 (*r_s_* = 0.478, *P* < .05), Asp f 4 (*r_s_* = 0.540, *P* < .05), and Asp f 6 (*r_s_* = 0.483, *P* < .05) were correlated with that of *P chrysogenum*, Asp f 2 (*r_s_* = 0.524, *P* < .05) and Asp f 6 (*r_s_* = 0.537, *P* < .05) was correlated with *M racemosus,* and Asp f 2 (*r_s_* = 0.568, *P* < .05) and Asp f 3 (*r_s_* = 0.514, *P* < .05) were correlated with *A alternata*; other mycotic allergens did not have significant correlation with *A fumigatus* components. Interestingly, the correlation between *A fumigatus* and its Asp f 2 component was the strongest (ABPA: *r_s_* = 0.786; Af‐sensitized asthma: *r_s_* = 0.663). Optimal scale analysis showed that ABPA was more relevant to Af components (Cronbach's alpha = 90.7%; Figure 4).

## 4 DISCUSSION

Although *A fumigatus*‐specific precipitins (Ouchterlony immunodiffusion test) have been widely used in the diagnosis of ABPA, their positive rates in patients with ABPA range widely from 27% to 87%.^20^ Moreover, their diagnostic value is limited. Currently, component‐resolved diagnosis (CRD) has been applied to the diagnosis of *A fumigatus* allergy, which is conducive to the accurate diagnosis of ABPA.

In our study, we found that 31.9% of patients were sensitized to *A fumigatus* in Class 3. Guangzhou city is influenced by the East Asian monsoon season and has a humid and warm subtropical climate, with a relative air humidity of 68% and annual precipitation of more than 1700 mm.^21^ This contributes greatly to the proliferation and growth of fungi, which prefer humid and warm environments. Therefore, a high concentration of mycotic spores in indoor and outdoor air is one of the most important causes of allergic respiratory tract diseases in Guangzhou.^22^, ^23^, ^24^

Interestingly, 94.44% of ABPA patients and 87.04% of Af‐sensitized asthma patients were co‐sensitized to at least one other fungal allergen. This is concurrent with previous reports by Chang et al and Ezeamuzie et al showing that *A fumigatus*, *C albicans*, and *P chrysogenum* were fungal allergens with the highest co‐sensitization rates among all allergenic fungi.^3^, ^5^ SIgE sensitization to fungal species is well reflected in their phylogenetic relationships, since IgE reactivity is more correlated in closely related molds than with phylogenetically distant molds.^25^, ^26^ This phenomenon suggests that there may be a cross‐reaction between allergies to *A fumigatus* and other fungi.

In a study conducted in Sweden in 2010, Soeria‐Atmadja et al analyzed the associations among mycotic allergies in 688 patients who were allergic to fungi. They reported that *A fumigatus* had extremely strong correlations with *P chrysogenum* and *H halodes* (*r_s_* = 0.85 and 0.87, respectively) and strong correlations with *C herbarum*, *C albicans*, and *A alternata* (0.60 ≤ *r_s_* < 0.80).^26^ However, in the present study, *A fumigatus* was strongly correlated with *C herbarum* (*r_s_* = 0.688) in ABPA and *A alternata* (*r_s_* = 0.692) in Af‐sensitized asthma patients but not correlated with *C albicans* (*P* > .05).

The difference between fungal and non‐fungal allergens is that fungal allergens are more complex. They contain proteases, glycosidases, and protein products, which can easily lead to cross‐reactions. Therefore, exposure to a single mycotic spore is equivalent to exposure to all fungal allergens.^27^ For example, *P chrysogenum* and *A fumigatus* both belong to family Trichocomaceae.^25^ Interaction between serum anti‐*P chrysogenum* antibodies in patients with ABPA can be greatly inhibited by *A fumigatus*, probably owing to the high similarity between the primary allergenic components of *P chrysogenum*, that is, alkaline and vacuolar serine proteases and their homologous allergenic components in *A fumigatus* (Asp f 13 and Asp f 18).^11^ Furthermore, the allergenic component in *A alternata*, manganese‐dependent superoxide dismutase (MnSOD), is the primary cause of the cross‐reaction with Asp f 6.^28^

In addition, ribosomal proteins are allergenic constituents of *A fumigatus* components (Asp f 8 and Asp f 23), *A alternata* components (Alt a 5 and Alt a 12), and *C herbarum* components (Cla h 5 and Cla h 12).^29^ Enolases are allergenic constituents of the *A fumigatus* component Asp f 22, *A alternata* component (Alt a 6), and *C herbarum* component (Cla h 6).^29^ Therefore, cross‐reactivity among fungal allergens should be considered when diagnosing fungal allergies to determine the appropriate treatment regimen.

Moreover, the positive rates of Asp f 1, Asp f 2, Asp f 4, and Asp f 6 in ABPA patients were significantly higher than those in Af‐sensitized asthma patients in our study. Patients with ABPA were characterized by higher levels of IgE antibodies to Asp f 1, Asp f 4, and Asp f 6 than those of Af‐sensitized asthma patients. A previous study showed that the combination of Asp f 1 and Asp f 2 can be considered a specific allergenic component in diagnosing *A fumigatus* sensitization.^12^ However, some other reports showed that the sIgE levels for Asp f 2, Asp f 4, and Asp f 6 were highly specific markers for ABPA diagnosis, with levels significantly higher in the serum of patients with ABPA than in the serum of Af‐sensitized asthma patients.^12^, ^29^ The combination of sensitized to all 5 component allergens of *A fumigatus* were higher in patients with ABPA (36.84%) than that in patients with asthma (18.52%). Therefore, in the presence of an *A fumigatus* allergy, in‐depth analysis of *A fumigatus* components could help to differentiate ABPA from Af‐sensitized asthma patients. The insufficient sample size, which was the main limitation of this study, should be supplemented by future follow‐up studies.

## 5 CONCLUSION

In summary, this study is the first to demonstrate the complex relationship between *A fumigatus* components and various mycotic allergens in ABPA and Af‐sensitized asthma patients from Guangzhou, Southern China. Asp f 1, Asp f 2, Asp f 4, and Asp f 6 in ABPA patients were significantly higher than those in Af‐sensitized asthma patients and were connected with various mycotic allergens. This finding is expected to help local doctors in the diagnosis of fungal allergies, particularly in differential diagnosis between ABPA and Af‐sensitized asthma.

## CONFLICT OF INTEREST

The authors declare that they have no competing interests.

## AUTHOR'S CONTRIBUTIONS

BQS conceived and designed the experiments. NLW, HMH, PYZ, and YL performed the experiments. WTL and ZHW analyzed the data. HSH and WTL wrote the article. All authors read and approved the final manuscript.

## ETHICAL STATEMENT

Approval was obtained from the ethics committee of The First Affiliated Hospital of Guangzhou Medical University (Reference number: GYFYY‐2016‐73).

## Data Availability

The data that support these findings are available on reasonable request from the corresponding author BQS. Data are not publicly available due to concerns regarding research participant privacy.
